# Wolcott–Rallison Syndrome: A Case Report with Novel Homozygous EIF2AK3 Gene Mutation

**DOI:** 10.1055/a-2800-2131

**Published:** 2026-03-26

**Authors:** Qiaomian Zhu, Xiaolin Wang, Huiping Zhang

**Affiliations:** 1 611822 Department of Neonatal Intensive Care Medicine, Xi’an Jiaotong University Affiliated Children’s Hospital, Xi’an, China; 2 74761 Department of Neonatology, Shanxi Medical University Second Affiliated Hospital, Taiyuan, China

## Introduction

Wolcott–Rallison syndrome (WRS, MIM #226980) is a rare autosomal recessive monogenic disorder. The disease was first reported by Wolcott and Rallison in 1972 (Wolcott CD et al., J Pediatr 1972; 80: 292–297), In 2000, Nicolino confirmed that the eukaryotic translation initiation factor 2α kinase 3 ( *EIF2AK3* ) gene was the pathogenic gene causing WRS (Delépine M et al., Nat Genet 2000; 25: 406–409). The condition is characterized by permanent diabetes presenting in early infancy or during the neonatal period, multiple epiphyseal dysplasia, liver failure, and growth retardation. Other clinical manifestations reported include mental retardation, renal failure, pancreatic exocrine dysfunction, neutropenia, hypothyroidism, and anemia (Asl SN et al., J Pediatr Endocrinol Metab 2019; 32: 607–613).

The *EIF2AK3* gene is located at 2p11.2, approximately 70 kb, and contains 17 exons, encoding EIF2AK3, also known as the protein kinase R-like endoplasmic reticulum kinase (PERK; Delépine M et al., Nat Genet 2000; 25: 406–409). PERK consists of 1,116 amino acid residues, including a regulatory domain and a catalytic domain, and plays a critical role in fetal and early pancreatic beta (β)-cell proliferation, differentiation, proinsulin processing, and stimulating bone growth (Senée V et al., Diabetes 2004; 53: 1876–1883). Endoplasmic reticulum (ER) stress occurs when misfolded or unfolded proteins in the ER exceed the processing capacity of the ER, leading to PERK autophosphorylation, which in turn phosphorylates the alpha subunit of eukaryotic initiation factor-2. This initiates the unfolded protein response, which reduces the synthesis of misfolded or unfolded proteins and upregulates activating transcription factor 4, which induces the expression of genes involved in antioxidant response, autophagy, amino acid metabolism, and apoptosis (Zhao N et al., Front Pediatr 2021; 9: 679646). *EIF2AK3* gene variants lead to impaired PERK phosphorylation, reduced ER ability to process stress, and accumulation of misfolded ER proteins, which cause pancreatic β cell defects and apoptosis, resulting in permanent neonatal diabetes and epiphyseal dysplasia (Fatani TH et al., BMC pediatr 2019; 19: 85).

Here, we report a case of WRS caused by a novel homozygous variation in the *EIF2AK3* gene presenting with an unusual feature.

## Case presentation

Our patient is a 7-week-old girl who was born by cesarean section at term, with a birth weight of 2300 g. She had healthy non-consanguineous parents. She had no abnormal birth history and was previously healthy.

Two days before admission, the child developed a mild cough, accompanied by irritability, poor response, refusal of feeding and decreased urine output. The patient went to a local hospital. Her blood gas analysis revealed metabolic acidosis, the venous blood glucose level was 62.17 mmol/L, the serum potassium level was 7.14 mmol/L, and the hemoglobin level was 68 g/L. She was diagnosed with diabetic ketoacidosis (DKA) and anemia, and she was referred to our hospital after receiving intravenous fluid therapy and rapid-acting intravenous insulin injection at the initial hospital.

Physical examination revealed that she had lethargy, moderate dehydration, mild jaundice and pallor, dry lips and tongue, sunken eyes and fontanel. Her breathing was deep and irregular, with normal lungs and heart by auscultation, her liver and spleen were not palpable, her abdomen was slightly flatulent, and her bowel sounds were weakened, with one to two times/min.

Laboratory tests showed elevated alanine aminotransferase (ALT), aspartate aminotransferase (AST), and direct bilirubin levels, along with decreased serum albumin in liver function tests. Other laboratory findings indicated kidney failure, central hypothyroidism, metabolic acidosis and neonatal diabetes ( Table 1 ). Urine analysis indicated that the patient had ketosis, glycosuria, hematuria and proteinuria. In coagulation tests, the international normalised ratio (INR) was as low as 1.16, while prothrombin time and activated partial thromboplastin time were within normal ranges. Renal color Doppler ultrasound showed an increased volume of both kidneys, enhanced parenchymal echo and an increased arterial resistance index of both kidneys. Skeletal survey did not show any bone abnormality.

**Table:** **Table 1** Laboratory results of the patient.

Text	Result	Reference range
ALT	352	8–71 U/L
AST	750	21–80 U/L
ALB	20.9	35–50 g/L
BUN	31.73	0.8–5.3 mmol/L
Cr	216	13–33 µmol/L
CK	8,000	41–330 U/L
CKMB	960	0–25 U/L
LDH	8,600	170–450 U/L
GLU	16.7	2.8–4.7 mmol/L
Insulin levels	1.13	2.6–24.9 µU/mL
Serum C-peptide levels	0.01	1.1–4.4 ng/mL
Hemoglobin A1c	8.03	3.6–6.0%
hs-CRP	42.29	0–3 mg/L
PCT	28.38	0–0.05 ng/mL
Plasma PH	6.935	7.35–7.45
HCO3 ^−^	5.9	22–26 mmol/L
BE	−26.03	−3–3 mmol/L
TSH	0.21	0.4–8.6 µIU/L
FT4	0.42	0.89–1.7 ng/dl
FT3	<1.07	1.71–4.87 pg/mL

Abbreviations: ALB, albumin; ALT, alanine aminotransferase; AST, aspartate aminotransferase; BUN, blood urea nitrogen; CK, creatine kinase; CKMB, Creatine kinase isoenzyme; Cr, serum creatinine; FT3, free triiodothyronine; FT4, free thyroxine; GLU, venous glucose; hs-CRP, hypersensitive C-reactive protein; LDH, lactic dehydrogenase; PCT, procalcitonin; TSH, thyroid-stimulating hormone.

The patient continued to receive short-acting insulin infusion (0.05U/kg/h) after admission to our hospital and a broad-spectrum antibiotics was started. Following the International Society of Pediatric and Adolescent Diabetes guidelines for DKA, the subsequent intravenous fluid composition was adjusted according to the blood sugar level. Gradually, blood glucose levels dropped and were maintained at 105–206 mg/dL. On the second day after admission, she developed anuria, and given the patient’s life-threatening renal failure and severe sepsis, we immediately performed continuous veno-venous hemodia-filtration.

On the third day of hospitalization, her condition deteriorated, her lethargy did not improve, and her abdominal distension progressed. Urgent abdominal ultrasonography and a supine abdominal X-ray were done, which confirmed necrotizing enterocolitis and intestinal perforation. We held a multi-disciplinary team meeting, including an endocrinologist, a nephrologist, and a neonatal surgeon, who agreed on performing an exploratory laparotomy immediately, and her parents were constantly involved in the decision-making process. Necrotizing enterocolitis and ileum perforation were eventually identified intraoperatively. After the surgery, all necessary measures were implemented in our neonatal intensive care medicine, and her kidney and liver functions improved. Her jaundice subsided, with ALT and AST levels decreasing compared to previous readings. The INR increased to 1.85. Her sensorium improved, and her voluntary movements increased. However, she developed persistent thrombocytopenia, with a minimum of 9×10 ^9^ /L, requiring repeated platelet transfusion therapy. Finally, her parents chose to give up on treatment due to uncertainty of prognosis, and she died after being discharged from the hospital against medical advice.

**Fig. 1:**
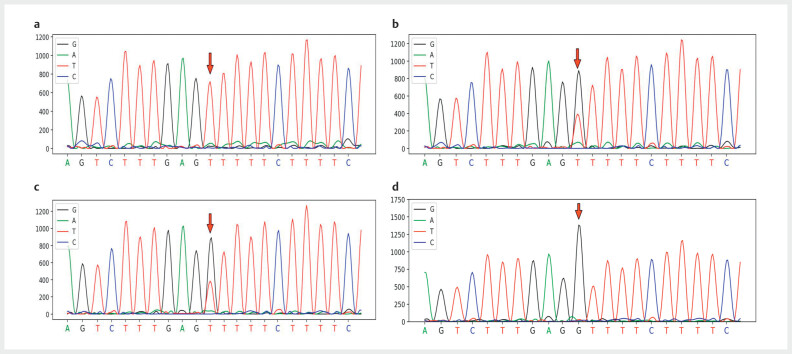
Sanger sequencing peak map of the *EIF2AK3* gene NM_004836: c.2685C>A (p.N895K) variation: ( **a** ) patient, ( **b** ) patient’s father, ( **c** ) patient’s mother, and ( **d** ) patient’s sister.

Given that she had permanent neonatal diabetes and DKA, she was advised to perform genetic analysis. Whole-exome-sequencing was done and a homozygous variant of *EIF2AK3* gene NM_004836: c.2685C>A (p.N895K) was identified ( Fig. 1 ). Both laboratory studies and genetic tests were conducted after obtaining an informed consent from the parents. The study was also approved for publication by the Departmental Review Board.

## Discussion

WRS is rare autosomal recessive disorder, which has been infrequently reported worldwide. Until December 2023, fewer than 120 variants have been reported as the disease causative factors of WRS in the Human Gene Mutation Database. Amongst the clinical features of WRS, diabetes usually appears within 6 months of birth and diabetes is often complicated by severe DKA at onset. However two patients have been diagnosed at ages 14 and 30 months (Senée V et al., Diabetes 2004; 53: 1876–1883). Multiple epiphyseal dysplasia often develops gradually 1–2 years after the diagnosis of diabetes and mainly affects the long bones, pelvis, and spine, but can also involve the cervical spine, leading to spinal cord compression (Dias RP et al., Orphanet J Rare Dis 2016; 11: 14). Affected children usually show slower rates of growth after 1 year of age and stagnation of height after 10 years of age (Senée V et al., Diabetes 2004; 53: 1876–1883). Hepatic failure is often recurrent and may resolve spontaneously, but in severe cases, patients may develop fulminant hepatitis which may be accompanied by multiple organ failure, leading to death (Julier C et al., Orphanet J Rare Dis 2010; 5: 29). These clinical manifestations of WRS are often precipitated by infection or stress, and the prognosis of children is often poor, as most of them die in infancy (Abbasi F et al., Can J Diabetes 2018; 42(3): 272–275).

We report a novel homozygous variant c.2685C > A (p.N895K) in the *EIF2AK3* gene in the newly diagnosed diabetic infant presenting with DKA. She had typical clinical features of WRS including permanent neonatal diabetes, acute hepatic failure, kidney failure, normocytic anemia, and central hypothyroidism. In addition, the child also developed necrotizing enterocolitis and ileum perforation. To the best of our knowledge, this clinical feature and this homozygous variant of the *EIF2AK3* gene have never been reported before. The pathogenesis of necrotizing enterocolitis and ileum perforation in this child is unknown. Increasing evidence shows that ER stress can cause apoptosis and damage intestinal mucosa, which is related to intestinal inflammation (Kaser A et al., Cell 2008; 134(5): 743–756). ER stress can be caused by an increase in protein synthesis which is stimulated by infections. Therefore, we suspect that the patient had activated ER stress as a result of the infection, and that this new mutation in the *EIF2AK3* gene leads to a reduced ability of the ER to process stress, causing increased ER stress in intestinal stem cells and resulting in apoptosis. It needs further cases with the same mutation or the same presentation to confirm.

Among the previously reported complications of WRS, hepatic failure is the most common life-threatening complication (Fatani TH et al., BMC pediatr 2019; 19: 85). Controlling blood glucose levels combined with organ transplantation are considered the best treatment to improve the survival rate of those patients. However, it is clear that our failure to anticipate the onset of her necrotizing enterocolitis and intestinal perforation led to worsening of her condition and poorer prognosis.

This case report enriches the genotype and phenotype spectrum of the disease and raises clinicians awareness of patients with WRS. It is suggested that testing for *EIF2AK3* gene mutation should be performed as early as possible in patients with permanent neonatal diabetes especially those with liver infection, even if the parents were non-consanguineous, because early diagnosis is of great value for predicting WRS co-morbidities and timely intervention to prevent life-threatening complications.

